# Crystal structure of tetra­methyl­tetra­thia­fulvalenium (1*S*)-camphor-10-sulfonate dihydrate

**DOI:** 10.1107/S2056989015010294

**Published:** 2015-06-03

**Authors:** Mathieu Sommer, Magali Allain, Cécile Mézière, Flavia Pop, Michel Giffard

**Affiliations:** a Université d’Angers, CNRS UMR 6200, Laboratoire MOLTECH-Anjou, 2 Bd Lavoisier, 49045 Angers, France

**Keywords:** crystal structure, tetra­thia­fulvalene-based materials, chirality, hydrogen bonding

## Abstract

Electro-oxidation of tetra­methyl­tetra­thia­fulvalene (TMTTF) in the presence of the chiral anion (1*S*)-camphor-10-sulfonate (S-camphSO_3_^−^) in tetra­hydro­furan/water medium afforded a 1/1 salt formulated as TMTTF·S-camphSO_3_·2H_2_O or 2-(4,5-dimethyl-1,3-di­thiol-2-yl­idene)-4,5-dimethyl-1,3-di­thiole radical ion (1+) [(1*S*)-7,7-dimethyl-2-oxobi­cyclo­[2.2.1]heptan-1-yl]methane­sulfonate dihydrate, C_10_H_12_S_4_^+^·C_10_H_15_O_4_S^−^·2H_2_O. In this salt, two independent TMTTF units are present but, in both cases, the observed bond lengths and especially the central C=C distance [1.392 (6) and 1.378 (6) Å] are in agreement with a complete oxidation of TMTTF which is thus present as TMTTF**^.^** ^+^ radical cations. These cations form one-dimensional stacks in which they are associated two by two, forming dimers with short [3.472 (1) to 3.554 (2) Å] S⋯S contacts. The two S-camphSO_3_ anions present also form stacks and are connected with each other *via* the water mol­ecules with many O—H⋯O hydrogen bonds ranging from 1.86 (3) to 2.15 (4) Å; the O—H⋯O hydrogen-bonding network can be described as being constituted of *C*_2_^2^(6) chains bearing *R*_3_^3^(11) lateral rings. On the other hand, the columns of cations and anions are connected through C—H⋯O hydrogen bonds, forming a system expanding in three directions; finally, the result is a three-dimensional network of O—H⋯O and C—H⋯O hydrogen bonds.

## Abstract 2

In this salt, two types of TMTTF units are present as TMTTF**^.^** ^+^ radical cations which form one-dimensional stacks in which they are associated two by two, forming dimers with short S⋯S contacts. The S-camphSO_3_ anions also form stacks and are connected with each other *via* O—H⋯O hydrogen bonds. The columns of cations and anions are connected through C—H⋯O hydrogen bonds.

## Chemical context   

Chiral mol­ecular conductors may display inter­esting properties such as the magneto-chiral anisotropy effect; the different strategies of access to these materials have been recently reviewed (Avarvari & Wallis, 2009 ▸; Pop *et al.*, 2014 ▸). Among these possible strategies, a straightforward *a priori* approach consists of combining, through electrocrystallization experiments, chiral counter-anions, existing in enanti­opure form, to TTF-based radical-cations; in this context, due to the commercial availability of the parent acid S-camphSO_3_H, the anion S-camphSO_3_^−^ appears to be a ready choice and, in fact, it has already been used to obtain the salt (EDT-TTFI_2_)_2_·S-camphSO_3_·H_2_O, where EDT-TTFI_2_ is di­iodo­ethyl­enedi­thiotetra­thia­fulvalene (Brezgunova *et al.*, 2010 ▸). In addition, it is worth mentioning a more general review relating to conducting radical cation salts with organic anions, especially anions derived from carb­oxy­lic and sulfonic organic acids (Geiser & Schlueter, 2004 ▸).
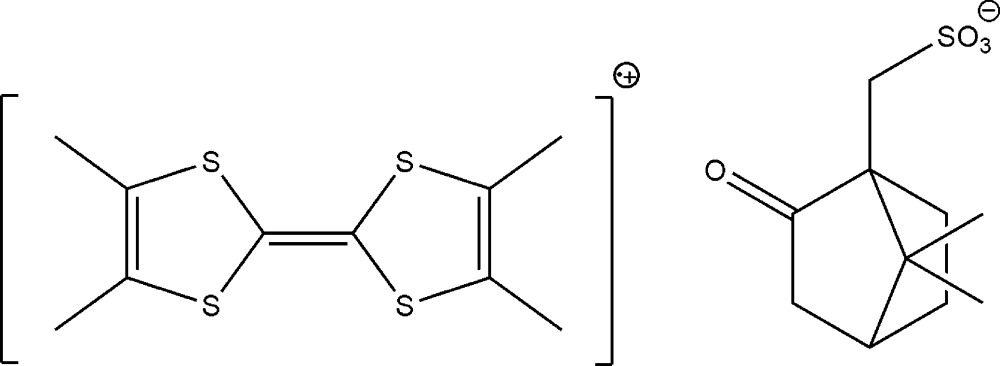


## Structural commentary   

**Figure 1:**
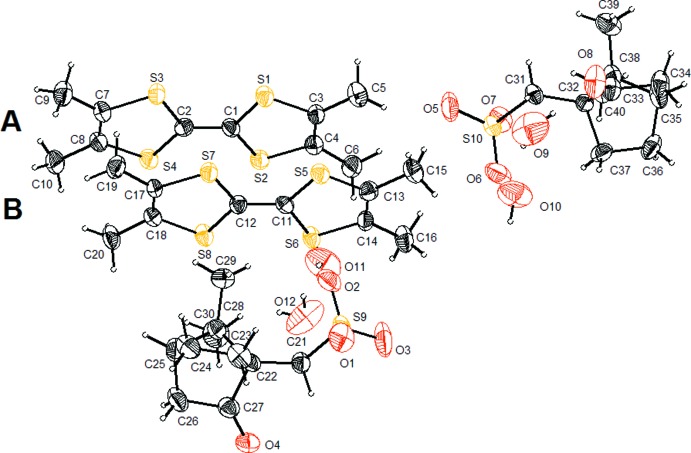
The asymmetric unit of the title compound, showing the atom-numbering scheme. Displacement ellipsoids are drawn at the 50% probability level. H atoms are represented as small spheres of arbitrary radii.

The title compound crystallizes with two independent TMTTF cations, two independent S-camphSO_3_ anions and four water mol­ecules (Fig. 1 ▸) in the asymmetric unit. The geometries of the two types (*A* and *B*) of TMTTF units (Fig. 1 ▸), are rather similar despite the fact that *A* and *B* are crystallographically independent; in both case, the observed bond lengths (see *e.g.* Penicaud *et al.*, 1990 ▸; Shibaeva, 1984 ▸) and especially the central C=C distance [1.392 (6) Å in *A* and 1.378 (6) Å in *B*] are in agreement with a complete oxidation of TMTTF which is thus present as TMTTF^.+^ radical-cations, in agreement also with the 1/1 cation/anion balance of this salt.

## Packing of the donors   

**Figure 2:**
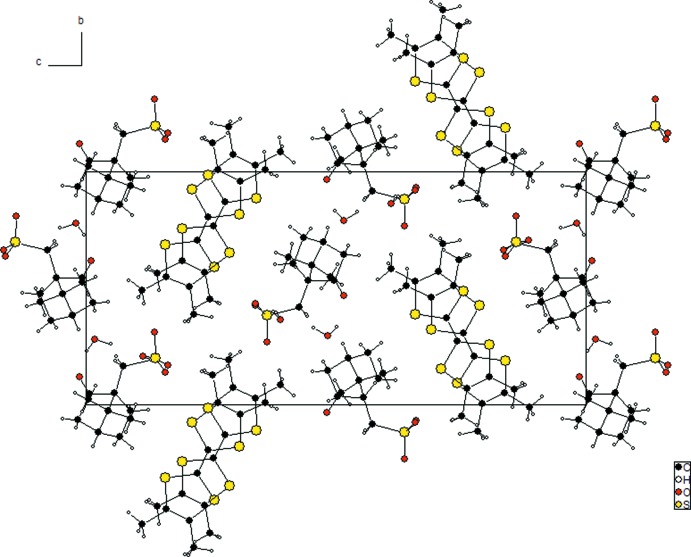
Overall view, along the *a* axis, of the crystal packing.

The cations form columns along the *a* axis in which the two types, *A* and *B*, of TMTTF units alternate (Fig. 2 ▸). The overall arrangement of the donors can be described as mono-dimensional since these stacks are isolated. Starting from one particular column, a set of equivalent columns may be deduced by translation along *b*, thus generating a cationic layer lying in the *ab* plane; however, there is no vicinity relation between two successive donors belonging to two different stacks of the same layer, except for proximity of the external methyl groups. When looking in the *c*-axis direction, successive layers are completely separated by slabs of anions; moreover, the orientation of the donors is different in two consecutive cationic layers since they adopt a herringbone arrangement.

**Figure 3:**
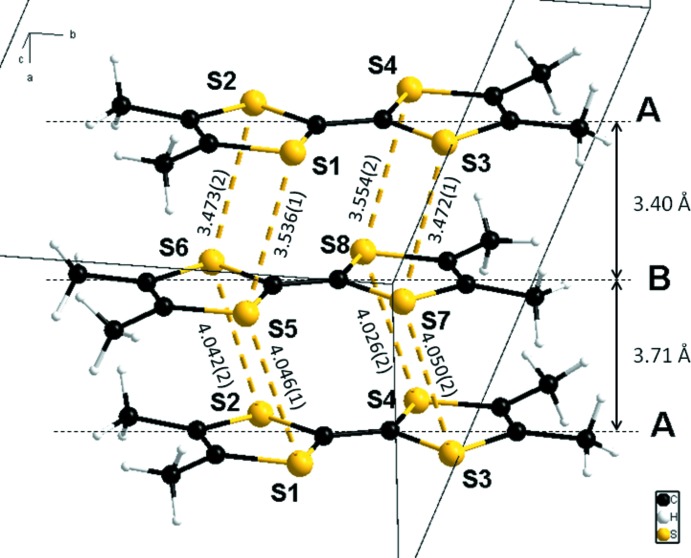
Packing of the donors: S⋯S contact distances within a stack, in the case of the two different inter-donor inter­vals.

The packing of the donors within one stack is shown more precisely in Fig. 3 ▸. The two alternating mol­ecules (*A* and *B*) are nearly parallel, the dihedral angle between their mean planes being only 0.24°. Within a stack, two independent inter­molecular inter­vals alternate with mean inter-plane distances of 3.40 and 3.71 Å. One can conclude in favour of the presence of dimers since the four inter­molecular S⋯S contacts corresponding to the smaller inter­val range from 3.472 (1) to 3.554 (2) Å (Fig. 3 ▸) and thus are shorter than twice the van der Waals radius of sulfur (3.6–3.7 Å: Bondi, 1964 ▸; Pauling, 1960 ▸); within a dimer the *A* and *B* units adopt a bond-over-ring (Williams *et al.*, 1992 ▸) relative arrangement. On the other hand, all S⋯S distances across the larger inter­val exceed the van der Waals distance, ranging from 4.026 (2) to 4.050 (2) Å.

## Supra­molecular features   

**Figure 4:**
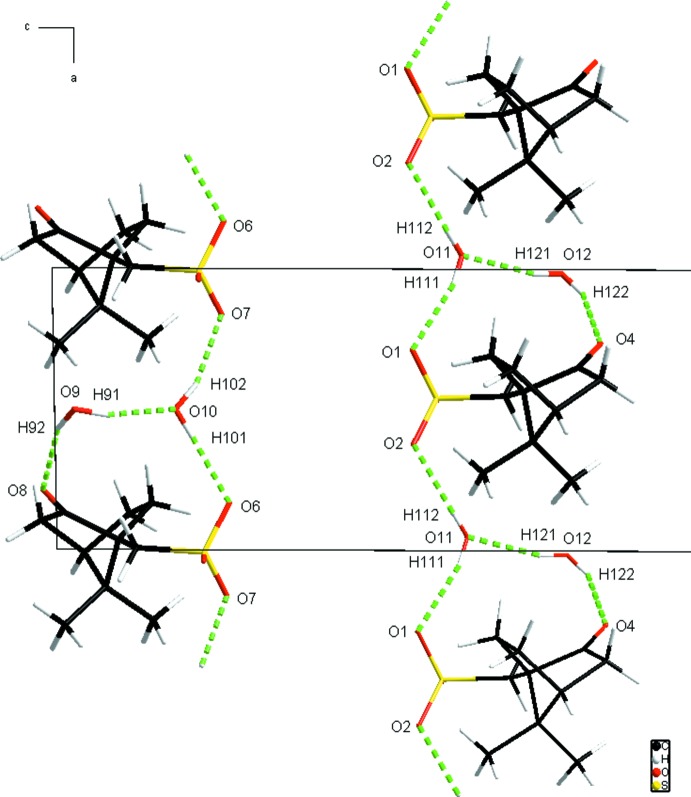
O—H⋯O hydrogen bonds (green dashed lines) between sulfonate anions and water mol­ecules (the TMTTF**^.^** ^+^ cations have been omitted for clarity).

**Figure 5:**
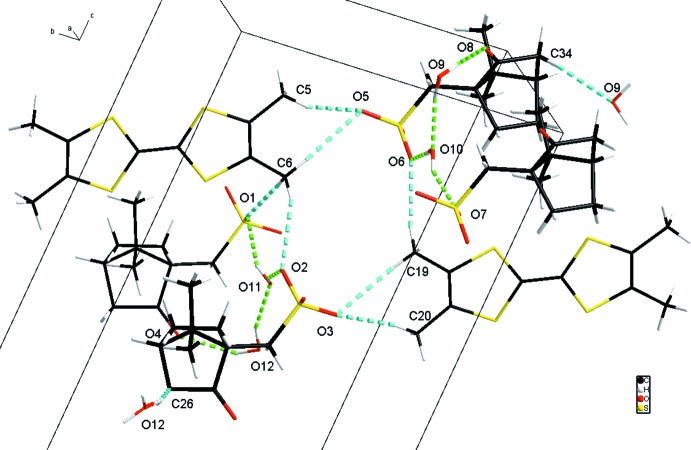
Partial view of the crystal packing, showing both O—H⋯O bonds (green dashed lines) and the C—H⋯O contacts (blue dashed lines).

The S-camphSO_3_ anions stack along the *a* axis and are connected with each other *via* the water mol­ecules with many O—H⋯O hydrogen bonds ranging from 1.86 (3) Å to 2.15 (4) Å (Table 1 ▸). The oxygen from one sulfonate is linked to the oxygen of the neighbouring sulfonate through a bridg­ing water mol­ecule, while the oxygen of this latter is linked to the H atom of another water mol­ecule, which is also connected to the oxygen of the ketone group, through O—H⋯O inter­actions (Fig. 4 ▸). Thus, in Etter’s classification (Etter, 1990 ▸), the O—H⋯O hydrogen-bonding network can be described as being constituted of 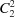
(6) chains bearing 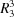
(11) lateral rings. On the other hand, the columns of cations and anions are connected through C—H⋯O hydrogen bonds, forming a system expanding in all three directions (Fig. 5 ▸ and Table 1 ▸); finally, the result is a three-dimensional network of O—H⋯O and C—H⋯O hydrogen bonds.

## Synthesis and crystallization   

**Synthesis of the supporting electrolyte** (1*S*)-camphor-10-sulfonic acid (Aldrich) (2.32 g, 10 mmol) was dissolved in water (50 ml), then 10 ml of a 1.0 mol l-1 methano­lic solution of tetra­butyl ammonium hydroxide (Aldrich) were added dropwise. This aqueous solution was stirred for one hour then extracted twice with di­chloro­methane (2 × 100 ml). After drying over MgSO4, evaporation of di­chloro­methane afforded tetra­butyl­ammonium *S*-camphorsulfonate (Bu_4_N^+^·S-camphSO_3_^−^) (4.50 g, yield 95%), m.p. 410–412 K. Elemental analysis: calculated for C_26_H_51_NO_4_S: C 65.92, H 10.85, N 2.96, S 6.77%; found: C 65.77, H 11.25, N 2.91, S 6.76%.^1^H NMR (300 MHz, CDCl3): δ 0.82 (3H, *s*), 1.00 (12H, *t*, *J* = 7.3 Hz), 1.15 (3H, s), 1.32 (1H, *m*), 1.45 (8H, pseudo sextuplet), 1.66 (8H, *m*), 1.83 (3H, pseudo *t*), 1.99 (2H, *m*), 2.29 (1H, *m*), 2.83 (2H, *m*), 3.31 (8H, pseudo *q*).

**Electrocrystallization of TMTTF·S-camphSO_3_·2H_2_O** A conventional H-shaped cell was charged with 142 mg (0.3 mmol) of Bu_4_N^+^·S-camphSO_3_^−^ dissolved in 20 ml of a 98/2 (*v*/*v*) tetra­hydro­furan–water mixture, degassed with argon. TMTTF (7.8 mg, 0.03 mmol) was introduced in the anodic arm and was then electro-oxidized under galvanostatic conditions with stepwise increases of the applied current (Anzai *et al.*, 1995 ▸): 0.5 µA for 3 days, then 1 µA for 4 days, 2 µA for 3 days and finally 5 µA for 8 days; afterwards, the black needles of TMTTF·S-camphSO_3_·2H_2_O, deposited at the platinum wire anode, were collected. The electrocrystallization was conducted at room temperature except during the 6 last days during which the cell was cooled to 283 K.

**Unsuccessful electrocrystallization experiments** Electrocrystallizations, using Bu_4_N^+^·S-camphSO_3_^−^ (or other camphSO_3_^−^ salts) as supporting electrolyte, were attempted, in various solvent conditions, with the following donors: TTF itself, BEDT-TTF, ethyl­enedi­thio­tetra­thia­fulvalene (EDT-TTF) and tetra­methyl­tetra­selena­fulvalene (TMTSF), without affording usable crystals. Thus, TMTTF·S-camphSO_3_·2H_2_O and (EDT-TTFI_2_)_2_·S-camphSO_3_·H_2_O (Brezgunova *et al.*, 2010 ▸), are presently the only known salts associating the camphorsulfonate anion to TTF donors.

## Refinement   

Crystal data, data collection and structure refinement details are summarized in Table 2 ▸. All H atoms attached to C were fixed geometrically and treated as riding with C—H = 0.96 Å (idealized methyl group, torsion angle from electron density), 0.97 Å (methyl­ene) or 0.98 Å (methine), with *U*_iso_(H) = 1.2*U*eq(C) or *U*_iso_(H) = 1.5*U*eq(meth­yl). The H atoms of the water mol­ecule were located in a difference electron density map and then refined as riding on their parent O atoms with *U*_iso_(H) = 1.5*U*eq(O).

## Supplementary Material

Crystal structure: contains datablock(s) global, I. DOI: 10.1107/S2056989015010294/bg2555sup1.cif

Structure factors: contains datablock(s) I. DOI: 10.1107/S2056989015010294/bg2555Isup2.hkl

CCDC reference: 1403700

Additional supporting information:  crystallographic information; 3D view; checkCIF report

## Figures and Tables

**Table 1:** Hydrogen-bond geometry (, )

*D*H*A*	*D*H	H*A*	*D* *A*	*D*H*A*
C5H5*A*O5	0.96	2.37	3.263(7)	155
C6H6*C*O2	0.96	2.57	3.472(5)	156
C6H6*A*O5	0.96	2.49	3.450(6)	173
C6H6*B*O1^i^	0.96	2.49	3.409(5)	161
C19H19*A*O3^ii^	0.96	2.48	3.420(6)	167
C19H19*B*O6^ii^	0.96	2.44	3.387(5)	169
C20H20*A*O3^ii^	0.96	2.34	3.287(7)	171
C26H26*A*O12^iii^	0.97	2.5	3.435(7)	162
C34H34*A*O9^iv^	0.97	2.52	3.476(6)	169
O9H91O10	0.89(2)	1.86(3)	2.729(7)	166(8)
O9H92O8	0.88(2)	2.13(3)	2.976(6)	160(6)
O10H101O6	0.85(2)	1.95(2)	2.795(5)	177(6)
O10H102O7^v^	0.84(2)	2.04(4)	2.815(5)	154(7)
O11H111O1^i^	0.86(2)	2.01(3)	2.821(5)	158(7)
O11H112O2	0.83(2)	2.06(3)	2.863(5)	162(7)
O12H121O11	0.87(2)	1.92(3)	2.732(7)	156(7)
O12H122O4^i^	0.88(2)	2.15(4)	2.965(6)	154(7)

Symmetry codes: (i) 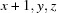; (ii) 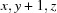; (iii) 
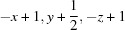
; (iv) 
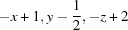
; (v) 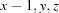.

**Table 2:** Experimental details

Crystal data	
Chemical formula	C_10_H_12_S_4_^+^C_10_H_15_O_4_S2H_2_O
*M*_r_	527.75
Crystal system, space group	Monoclinic, *P*2_1_
Temperature (K)	293
*a*, *b*, *c* ()	7.1612(6), 12.537(2), 26.906(4)
()	91.331(8)
*V* (^3^)	2415.0(6)
*Z*	4
Radiation type	Mo *K*
(mm^1^)	0.51
Crystal size (mm)	0.32 0.07 0.05

Data collection	
Diffractometer	Nonius KappaCCD
Absorption correction	Multi-scan (*SADABS*; Bruker, 2008 ▸)
*T*_min_, *T*_max_	0.818, 0.975
No. of measured, independent and observed [*I* > 2(*I*)] reflections	27748, 9990, 7458
*R*_int_	0.045
(sin /)_max_ (^1^)	0.661

Refinement	
*R*[*F* ^2^ > 2(*F* ^2^)], *wR*(*F* ^2^), *S*	0.042, 0.107, 1.09
No. of reflections	9990
No. of parameters	596
No. of restraints	14
H-atom treatment	H atoms treated by a mixture of independent and constrained refinement
_max_, _min_ (e ^3^)	0.31, 0.33
Absolute structure	Flack (1983 ▸), 4218 Friedel pairs
Absolute structure parameter	0.15(6)

Computer programs: *COLLECT* (Hooft, 2008 ▸), *DIRAX* (Duisenberg, 1992 ▸), *EVALCCD* (Duisenberg *et al.*, 2000 ▸), *SHELXS97* and *SHELXL97* (Sheldrick, 2008 ▸), *DIAMOND* (Brandenburg, 2014 ▸) and *WinGX* (Farrugia, 2012 ▸).

## supplementary crystallographic information

### Crystal data

**Table:**

C_10_H_12_S_4_^+^·C_10_H_15_O_4_S^−^·2H_2_O	*F*(000) = 1116
*M**_r_* = 527.75	*D*_x_ = 1.452 Mg m^−^^3^
Monoclinic, *P*2_1_	Mo *K*α radiation, λ = 0.71073 Å
Hall symbol: P 2yb	Cell parameters from 14430 reflections
*a* = 7.1612 (6) Å	θ = 2.3–28.0°
*b* = 12.537 (2) Å	µ = 0.51 mm^−^^1^
*c* = 26.906 (4) Å	*T* = 293 K
β = 91.331 (8)°	Needle, black
*V* = 2415.0 (6) Å^3^	0.32 × 0.07 × 0.05 mm
*Z* = 4	

### Data collection

**Table:**

Nonius KappaCCD diffractometer	9990 independent reflections
Graphite monochromator	7458 reflections with *I* > 2σ(*I*)
Detector resolution: 9 pixels mm^-1^	*R*_int_ = 0.045
CCD scans	θ_max_ = 28.0°, θ_min_ = 2.3°
Absorption correction: multi-scan (*SADABS*; Bruker, 2008)	*h* = −9→9
*T*_min_ = 0.818, *T*_max_ = 0.975	*k* = −16→16
27748 measured reflections	*l* = −34→35

### Refinement

**Table:**

Refinement on *F*^2^	Secondary atom site location: difference Fourier map
Least-squares matrix: full	Hydrogen site location: inferred from neighbouring sites
*R*[*F*^2^ > 2σ(*F*^2^)] = 0.042	H atoms treated by a mixture of independent and constrained refinement
*wR*(*F*^2^) = 0.107	*w* = 1/[σ^2^(*F*_o_^2^) + (0.0443*P*)^2^ + 0.7423*P*] where *P* = (*F*_o_^2^ + 2*F*_c_^2^)/3
*S* = 1.09	(Δ/σ)_max_ = 0.001
9990 reflections	Δρ_max_ = 0.31 e Å^−^^3^
596 parameters	Δρ_min_ = −0.33 e Å^−^^3^
14 restraints	Absolute structure: Flack (1983), 4218 Friedel pairs
Primary atom site location: structure-invariant direct methods	Absolute structure parameter: 0.15 (6)

### Special details

**Table:**

Geometry. All e.s.d.'s (except the e.s.d. in the dihedral angle between two l.s. planes) are estimated using the full covariance matrix. The cell e.s.d.'s are taken into account individually in the estimation of e.s.d.'s in distances, angles and torsion angles; correlations between e.s.d.'s in cell parameters are only used when they are defined by crystal symmetry. An approximate (isotropic) treatment of cell e.s.d.'s is used for estimating e.s.d.'s involving l.s. planes.
Refinement. Refinement of *F*^2^ against ALL reflections. The weighted *R*-factor *wR* and goodness of fit *S* are based on *F*^2^, conventional *R*-factors *R* are based on *F*, with *F* set to zero for negative *F*^2^. The threshold expression of *F*^2^ > σ(*F*^2^) is used only for calculating *R*-factors(gt) *etc*. and is not relevant to the choice of reflections for refinement. *R*-factors based on *F*^2^ are statistically about twice as large as those based on *F*, and *R*- factors based on ALL data will be even larger. The H atoms on the water molecules were added by Fourier difference map and then restrained with 13 *DFIX* commands between O and H and H and H on the 4 water molecules.

### Fractional atomic coordinates and isotropic or equivalent isotropic displacement parameters (Å^2^)

**Table:**

	*x*	*y*	*z*	*U*_iso_*/*U*_eq_	
C1	1.0039 (5)	0.7691 (4)	0.74395 (15)	0.0315 (10)	
C2	1.0143 (5)	0.8691 (4)	0.72164 (15)	0.0305 (10)	
C3	0.9649 (5)	0.6176 (4)	0.80764 (14)	0.0321 (10)	
C4	0.9783 (5)	0.5681 (4)	0.76277 (16)	0.0339 (10)	
C5	0.9429 (6)	0.5649 (4)	0.85764 (16)	0.0434 (12)	
H5A	0.9354	0.489	0.8534	0.065*	
H5B	0.8308	0.5902	0.8726	0.065*	
H5C	1.0485	0.582	0.8788	0.065*	
C6	0.9712 (6)	0.4506 (4)	0.75210 (17)	0.0441 (12)	
H6A	0.9787	0.4117	0.7828	0.066*	
H6B	1.0744	0.4313	0.7318	0.066*	
H6C	0.8561	0.4338	0.7349	0.066*	
C7	1.0197 (5)	1.0716 (4)	0.70563 (15)	0.0352 (11)	
C8	1.0332 (5)	1.0260 (4)	0.66036 (15)	0.0349 (11)	
C9	1.0103 (6)	1.1901 (4)	0.71473 (17)	0.0463 (12)	
H9A	1.0895	1.2264	0.6919	0.069*	
H9B	1.0515	1.2053	0.7482	0.069*	
H9C	0.8839	1.2142	0.7099	0.069*	
C10	1.0429 (7)	1.0846 (4)	0.61183 (16)	0.0506 (13)	
H10A	1.1688	1.1079	0.6068	0.076*	
H10B	0.9615	1.1454	0.6124	0.076*	
H10C	1.0047	1.038	0.5852	0.076*	
C11	0.4647 (5)	0.7052 (4)	0.77718 (14)	0.0290 (10)	
C12	0.4751 (5)	0.8039 (4)	0.75490 (15)	0.0295 (9)	
C13	0.4408 (5)	0.5506 (4)	0.83837 (15)	0.0346 (10)	
C14	0.4550 (5)	0.5035 (4)	0.79301 (15)	0.0312 (10)	
C15	0.4332 (6)	0.4884 (5)	0.88686 (15)	0.0450 (12)	
H15A	0.5534	0.4577	0.8943	0.067*	
H15B	0.3417	0.4327	0.8836	0.067*	
H15C	0.3995	0.5356	0.9133	0.067*	
C16	0.4630 (7)	0.3864 (4)	0.78380 (17)	0.0477 (13)	
H16A	0.3503	0.3537	0.7949	0.072*	
H16B	0.5682	0.3566	0.8017	0.072*	
H16C	0.4758	0.3735	0.7489	0.072*	
C17	0.4983 (5)	1.0066 (4)	0.73563 (15)	0.0313 (10)	
C18	0.5138 (5)	0.9570 (4)	0.69180 (13)	0.0302 (10)	
C19	0.5022 (6)	1.1239 (4)	0.74642 (16)	0.0432 (12)	
H19A	0.5142	1.1627	0.7159	0.065*	
H19B	0.6064	1.1398	0.7682	0.065*	
H19C	0.3883	1.1442	0.762	0.065*	
C20	0.5357 (6)	1.0069 (4)	0.64138 (15)	0.0429 (12)	
H20A	0.5225	1.0829	0.644	0.064*	
H20B	0.4415	0.9795	0.6189	0.064*	
H20C	0.657	0.9902	0.6291	0.064*	
C21	0.4508 (6)	0.4156 (3)	0.57341 (14)	0.0368 (10)	
H21A	0.5631	0.387	0.559	0.044*	
H21B	0.3465	0.3769	0.5585	0.044*	
C22	0.4333 (5)	0.5309 (3)	0.55588 (12)	0.0312 (8)	
C23	0.3084 (6)	0.6086 (4)	0.58581 (15)	0.0450 (11)	
H23A	0.177	0.5928	0.5802	0.054*	
H23B	0.3373	0.6041	0.6211	0.054*	
C24	0.3572 (6)	0.7193 (3)	0.56544 (15)	0.0518 (11)	
H24A	0.2471	0.7541	0.5514	0.062*	
H24B	0.4107	0.7642	0.5914	0.062*	
C25	0.4994 (6)	0.6970 (4)	0.52537 (15)	0.0442 (11)	
H25	0.5759	0.7583	0.516	0.053*	
C26	0.3896 (7)	0.6448 (3)	0.48247 (13)	0.0508 (11)	
H26A	0.2756	0.6837	0.4747	0.061*	
H26B	0.464	0.64	0.4529	0.061*	
C27	0.3477 (5)	0.5348 (4)	0.50338 (13)	0.0400 (9)	
C28	0.6108 (5)	0.6007 (3)	0.54804 (12)	0.0356 (9)	
C29	0.7228 (6)	0.6330 (4)	0.59547 (15)	0.0498 (12)	
H29A	0.804	0.6915	0.588	0.075*	
H29B	0.796	0.5734	0.607	0.075*	
H29C	0.6381	0.6541	0.6208	0.075*	
C30	0.7494 (6)	0.5486 (4)	0.51178 (15)	0.0585 (13)	
H30A	0.6807	0.513	0.4856	0.088*	
H30B	0.826	0.4979	0.5295	0.088*	
H30C	0.8271	0.6028	0.4978	0.088*	
C31	0.9937 (5)	0.1623 (3)	0.92505 (13)	0.0320 (9)	
H31A	1.108	0.187	0.9413	0.038*	
H31B	0.8927	0.2041	0.9385	0.038*	
C32	0.9641 (5)	0.0474 (3)	0.94173 (11)	0.0289 (8)	
C33	0.8680 (5)	0.0448 (3)	0.99285 (13)	0.0391 (9)	
C34	0.8975 (6)	−0.0648 (3)	1.01455 (14)	0.0459 (10)	
H34A	0.7797	−0.1012	1.0193	0.055*	
H34B	0.9666	−0.0618	1.0459	0.055*	
C35	1.0107 (6)	−0.1182 (4)	0.97438 (15)	0.0432 (11)	
H35	1.0812	−0.181	0.9857	0.052*	
C36	0.8726 (6)	−0.1400 (3)	0.93109 (14)	0.0493 (11)	
H36A	0.7588	−0.1724	0.9428	0.059*	
H36B	0.9274	−0.1862	0.9065	0.059*	
C37	0.8346 (6)	−0.0278 (4)	0.90965 (15)	0.0455 (10)	
H37A	0.7043	−0.0083	0.9129	0.055*	
H37B	0.8663	−0.0244	0.8748	0.055*	
C38	1.1338 (5)	−0.0259 (3)	0.95488 (13)	0.0371 (8)	
C39	1.2680 (6)	0.0219 (4)	0.99411 (17)	0.0598 (13)	
H39A	1.3389	0.0782	0.9794	0.09*	
H39B	1.3515	−0.0326	1.0063	0.09*	
H39C	1.1979	0.0499	1.0211	0.09*	
C40	1.2493 (6)	−0.0604 (4)	0.91036 (17)	0.0550 (13)	
H40A	1.3261	−0.1202	0.9196	0.082*	
H40B	1.3272	−0.0023	0.9003	0.082*	
H40C	1.167	−0.0802	0.8833	0.082*	
O1	0.2874 (4)	0.4218 (3)	0.66027 (11)	0.0628 (10)	
O2	0.6222 (4)	0.4306 (3)	0.66083 (10)	0.0534 (9)	
O3	0.4668 (6)	0.2661 (4)	0.63816 (14)	0.0813 (13)	
O4	0.2623 (4)	0.4644 (3)	0.48213 (10)	0.0586 (8)	
O5	1.0396 (5)	0.3107 (3)	0.86141 (12)	0.0650 (10)	
O6	0.8332 (4)	0.1656 (3)	0.83627 (11)	0.0575 (9)	
O7	1.1630 (4)	0.1376 (3)	0.84020 (10)	0.0476 (8)	
O8	0.7881 (4)	0.1182 (2)	1.01231 (10)	0.0581 (8)	
O9	0.5021 (7)	0.2784 (4)	0.9811 (2)	0.1014 (17)	
H91	0.522 (11)	0.258 (6)	0.9499 (10)	0.152*	
H92	0.570 (10)	0.230 (5)	0.997 (2)	0.152*	
O10	0.5008 (5)	0.2052 (5)	0.88554 (15)	0.0837 (13)	
H101	0.599 (5)	0.191 (6)	0.870 (2)	0.126*	
H102	0.410 (5)	0.200 (6)	0.8652 (17)	0.126*	
O11	0.9508 (5)	0.3613 (4)	0.61152 (16)	0.0889 (15)	
H111	1.053 (4)	0.393 (5)	0.6200 (16)	0.133*	
H112	0.865 (5)	0.395 (5)	0.625 (2)	0.133*	
O12	1.0154 (7)	0.2917 (5)	0.5172 (2)	0.1124 (19)	
H121	1.014 (12)	0.329 (6)	0.5444 (17)	0.169*	
H122	1.085 (10)	0.331 (6)	0.498 (2)	0.169*	
S1	0.97592 (14)	0.75623 (9)	0.80709 (4)	0.0348 (3)	
S2	1.01099 (15)	0.65135 (9)	0.71188 (4)	0.0375 (3)	
S3	1.00760 (14)	0.98512 (9)	0.75585 (4)	0.0337 (3)	
S4	1.03468 (15)	0.88716 (10)	0.65885 (4)	0.0380 (3)	
S5	0.44108 (14)	0.68713 (9)	0.84015 (4)	0.0350 (3)	
S6	0.47203 (15)	0.58999 (9)	0.74292 (4)	0.0345 (3)	
S7	0.46796 (15)	0.92346 (9)	0.78738 (4)	0.0349 (3)	
S8	0.50273 (15)	0.81870 (10)	0.69212 (4)	0.0360 (3)	
S9	0.45931 (16)	0.38087 (11)	0.63808 (4)	0.0435 (3)	
S10	1.00831 (14)	0.19673 (10)	0.86075 (4)	0.0361 (3)	

### Atomic displacement parameters (Å^2^)

**Table:**

	*U*^11^	*U*^22^	*U*^33^	*U*^12^	*U*^13^	*U*^23^
C1	0.039 (2)	0.032 (3)	0.023 (2)	0.0003 (19)	−0.0012 (15)	0.0016 (19)
C2	0.035 (2)	0.027 (3)	0.029 (2)	−0.0003 (18)	0.0003 (15)	−0.005 (2)
C3	0.035 (2)	0.027 (3)	0.034 (2)	0.0007 (18)	−0.0001 (16)	0.005 (2)
C4	0.037 (2)	0.031 (3)	0.034 (2)	0.0006 (19)	−0.0010 (16)	0.002 (2)
C5	0.050 (3)	0.043 (3)	0.038 (3)	0.008 (2)	0.0076 (19)	0.007 (2)
C6	0.055 (3)	0.032 (3)	0.044 (3)	0.000 (2)	−0.0025 (19)	−0.001 (2)
C7	0.040 (2)	0.035 (3)	0.031 (2)	0.0008 (19)	0.0015 (16)	0.007 (2)
C8	0.034 (2)	0.036 (3)	0.035 (2)	−0.0006 (18)	−0.0031 (16)	0.008 (2)
C9	0.062 (3)	0.032 (3)	0.044 (3)	0.000 (2)	0.007 (2)	0.002 (2)
C10	0.071 (3)	0.048 (4)	0.034 (3)	−0.014 (3)	0.000 (2)	0.012 (2)
C11	0.0312 (19)	0.033 (3)	0.023 (2)	0.0032 (19)	0.0019 (14)	0.002 (2)
C12	0.035 (2)	0.024 (3)	0.030 (2)	0.0026 (18)	0.0024 (15)	−0.0009 (19)
C13	0.035 (2)	0.038 (3)	0.032 (2)	−0.0039 (19)	0.0056 (16)	0.002 (2)
C14	0.033 (2)	0.028 (3)	0.033 (2)	−0.0019 (17)	0.0026 (16)	0.0068 (19)
C15	0.050 (2)	0.050 (3)	0.034 (2)	0.003 (2)	0.0046 (18)	0.011 (2)
C16	0.072 (3)	0.030 (3)	0.042 (3)	0.011 (2)	0.007 (2)	0.006 (2)
C17	0.039 (2)	0.026 (3)	0.028 (2)	−0.0015 (18)	−0.0031 (16)	0.0059 (19)
C18	0.0316 (19)	0.033 (3)	0.026 (2)	0.0023 (17)	0.0012 (15)	0.0036 (19)
C19	0.059 (3)	0.028 (3)	0.042 (3)	−0.006 (2)	0.003 (2)	−0.001 (2)
C20	0.060 (3)	0.041 (3)	0.029 (2)	0.000 (2)	0.0070 (18)	0.011 (2)
C21	0.051 (2)	0.030 (3)	0.029 (2)	0.000 (2)	0.0015 (16)	−0.0003 (18)
C22	0.038 (2)	0.034 (2)	0.0219 (16)	0.0023 (18)	−0.0015 (14)	−0.0006 (15)
C23	0.048 (2)	0.051 (3)	0.037 (2)	0.016 (2)	0.0088 (17)	−0.0042 (19)
C24	0.072 (3)	0.042 (3)	0.042 (2)	0.018 (2)	−0.008 (2)	−0.0084 (19)
C25	0.069 (3)	0.029 (3)	0.035 (2)	−0.005 (2)	0.001 (2)	0.003 (2)
C26	0.081 (3)	0.042 (3)	0.0291 (18)	0.008 (2)	−0.0063 (19)	0.0053 (17)
C27	0.046 (2)	0.041 (3)	0.0330 (18)	0.003 (2)	−0.0017 (16)	−0.0019 (18)
C28	0.041 (2)	0.036 (2)	0.0290 (17)	−0.0021 (18)	0.0007 (15)	0.0029 (16)
C29	0.053 (3)	0.049 (3)	0.046 (2)	−0.005 (2)	−0.0122 (19)	−0.002 (2)
C30	0.058 (3)	0.073 (4)	0.045 (2)	−0.002 (3)	0.019 (2)	−0.001 (2)
C31	0.040 (2)	0.035 (3)	0.0213 (17)	0.0033 (17)	0.0035 (14)	0.0031 (16)
C32	0.0349 (19)	0.031 (2)	0.0204 (15)	−0.0019 (17)	0.0016 (14)	−0.0006 (14)
C33	0.048 (2)	0.037 (2)	0.0333 (18)	0.001 (2)	0.0107 (17)	0.0007 (17)
C34	0.068 (3)	0.037 (3)	0.0335 (19)	−0.001 (2)	0.0188 (18)	0.0046 (17)
C35	0.067 (3)	0.033 (3)	0.030 (2)	0.007 (2)	0.0087 (19)	0.005 (2)
C36	0.073 (3)	0.031 (2)	0.044 (2)	−0.018 (2)	0.012 (2)	−0.0080 (18)
C37	0.056 (2)	0.043 (3)	0.037 (2)	−0.019 (2)	−0.0051 (18)	−0.0048 (19)
C38	0.046 (2)	0.030 (2)	0.0348 (18)	0.0075 (18)	0.0007 (16)	0.0054 (16)
C39	0.059 (3)	0.061 (4)	0.059 (3)	0.007 (3)	−0.020 (2)	0.005 (2)
C40	0.054 (3)	0.048 (3)	0.064 (3)	0.017 (2)	0.026 (2)	0.011 (2)
O1	0.0554 (18)	0.086 (3)	0.0478 (18)	−0.0072 (17)	0.0242 (14)	0.0106 (17)
O2	0.0572 (18)	0.069 (2)	0.0338 (15)	0.0003 (16)	−0.0061 (13)	0.0089 (15)
O3	0.153 (4)	0.035 (3)	0.057 (3)	0.004 (2)	0.006 (2)	0.017 (2)
O4	0.083 (2)	0.052 (2)	0.0404 (15)	−0.0110 (17)	−0.0206 (15)	−0.0034 (14)
O5	0.114 (3)	0.035 (2)	0.046 (2)	−0.006 (2)	0.0016 (19)	0.0137 (18)
O6	0.0423 (16)	0.085 (3)	0.0447 (16)	−0.0022 (16)	−0.0117 (13)	0.0147 (17)
O7	0.0464 (16)	0.061 (2)	0.0358 (15)	0.0008 (15)	0.0150 (12)	0.0057 (15)
O8	0.081 (2)	0.0403 (18)	0.0541 (17)	0.0092 (16)	0.0368 (15)	−0.0004 (14)
O9	0.092 (3)	0.075 (4)	0.137 (5)	0.021 (2)	−0.002 (3)	−0.035 (3)
O10	0.0447 (18)	0.126 (4)	0.080 (3)	0.003 (3)	−0.0029 (17)	0.007 (3)
O11	0.060 (2)	0.124 (4)	0.083 (3)	0.028 (3)	0.003 (2)	0.001 (3)
O12	0.100 (3)	0.095 (5)	0.143 (5)	−0.034 (3)	0.020 (3)	−0.035 (4)
S1	0.0475 (6)	0.0306 (7)	0.0266 (5)	0.0005 (5)	0.0046 (4)	0.0000 (5)
S2	0.0555 (6)	0.0308 (8)	0.0260 (5)	−0.0030 (5)	−0.0029 (4)	−0.0021 (5)
S3	0.0473 (6)	0.0258 (7)	0.0281 (6)	−0.0005 (5)	0.0048 (4)	0.0010 (5)
S4	0.0536 (6)	0.0345 (8)	0.0257 (5)	−0.0040 (5)	−0.0010 (4)	0.0006 (5)
S5	0.0475 (6)	0.0333 (8)	0.0240 (5)	−0.0009 (5)	0.0015 (4)	0.0001 (5)
S6	0.0491 (6)	0.0283 (7)	0.0263 (5)	0.0035 (5)	0.0060 (4)	0.0008 (5)
S7	0.0533 (6)	0.0274 (7)	0.0239 (5)	−0.0028 (5)	0.0011 (4)	−0.0010 (5)
S8	0.0535 (6)	0.0315 (7)	0.0233 (5)	0.0049 (5)	0.0064 (4)	−0.0003 (5)
S9	0.0629 (7)	0.0408 (8)	0.0269 (5)	−0.0014 (6)	0.0041 (5)	0.0078 (5)
S10	0.0428 (5)	0.0381 (8)	0.0274 (5)	−0.0013 (5)	0.0010 (4)	0.0085 (5)

### Geometric parameters (Å, º)

**Table:**

C1—C2	1.392 (6)	C23—H23A	0.97
C1—S2	1.711 (5)	C23—H23B	0.97
C1—S1	1.723 (4)	C24—C25	1.526 (6)
C2—S4	1.714 (4)	C24—H24A	0.97
C2—S3	1.723 (5)	C24—H24B	0.97
C3—C4	1.363 (6)	C25—C26	1.528 (6)
C3—C5	1.510 (6)	C25—C28	1.562 (6)
C3—S1	1.740 (5)	C25—H25	0.98
C4—C6	1.501 (7)	C26—C27	1.522 (6)
C4—S2	1.742 (4)	C26—H26A	0.97
C5—H5A	0.96	C26—H26B	0.97
C5—H5B	0.96	C27—O4	1.210 (5)
C5—H5C	0.96	C28—C29	1.546 (5)
C6—H6A	0.96	C28—C30	1.552 (5)
C6—H6B	0.96	C29—H29A	0.96
C6—H6C	0.96	C29—H29B	0.96
C7—C8	1.351 (6)	C29—H29C	0.96
C7—C9	1.507 (7)	C30—H30A	0.96
C7—S3	1.736 (4)	C30—H30B	0.96
C8—C10	1.502 (6)	C30—H30C	0.96
C8—S4	1.741 (5)	C31—C32	1.526 (6)
C9—H9A	0.96	C31—S10	1.789 (4)
C9—H9B	0.96	C31—H31A	0.97
C9—H9C	0.96	C31—H31B	0.97
C10—H10A	0.96	C32—C33	1.553 (4)
C10—H10B	0.96	C32—C38	1.557 (5)
C10—H10C	0.96	C32—C37	1.567 (5)
C11—C12	1.378 (6)	C33—O8	1.209 (5)
C11—S6	1.714 (5)	C33—C34	1.506 (6)
C11—S5	1.722 (4)	C34—C35	1.522 (5)
C12—S8	1.715 (4)	C34—H34A	0.97
C12—S7	1.736 (5)	C34—H34B	0.97
C13—C14	1.361 (6)	C35—C36	1.535 (6)
C13—C15	1.522 (6)	C35—C38	1.554 (6)
C13—S5	1.713 (5)	C35—H35	0.98
C14—C16	1.490 (7)	C36—C37	1.542 (6)
C14—S6	1.736 (4)	C36—H36A	0.97
C15—H15A	0.96	C36—H36B	0.97
C15—H15B	0.96	C37—H37A	0.97
C15—H15C	0.96	C37—H37B	0.97
C16—H16A	0.96	C38—C39	1.533 (6)
C16—H16B	0.96	C38—C40	1.534 (5)
C16—H16C	0.96	C39—H39A	0.96
C17—C18	1.340 (6)	C39—H39B	0.96
C17—C19	1.499 (6)	C39—H39C	0.96
C17—S7	1.757 (4)	C40—H40A	0.96
C18—C20	1.505 (5)	C40—H40B	0.96
C18—S8	1.736 (5)	C40—H40C	0.96
C19—H19A	0.96	O1—S9	1.473 (3)
C19—H19B	0.96	O2—S9	1.446 (3)
C19—H19C	0.96	O3—S9	1.440 (5)
C20—H20A	0.96	O5—S10	1.446 (4)
C20—H20B	0.96	O6—S10	1.456 (3)
C20—H20C	0.96	O7—S10	1.453 (3)
C21—C22	1.524 (6)	O9—H91	0.89 (2)
C21—S9	1.793 (4)	O9—H92	0.88 (2)
C21—H21A	0.97	O10—H101	0.846 (19)
C21—H21B	0.97	O10—H102	0.841 (19)
C22—C27	1.527 (5)	O11—H111	0.857 (19)
C22—C23	1.560 (5)	O11—H112	0.83 (2)
C22—C28	1.562 (5)	O12—H121	0.867 (19)
C23—C24	1.535 (6)	O12—H122	0.88 (2)
			
C2—C1—S2	123.8 (3)	C24—C25—H25	115
C2—C1—S1	121.2 (3)	C26—C25—H25	115
S2—C1—S1	115.0 (3)	C28—C25—H25	115
C1—C2—S4	123.4 (3)	C27—C26—C25	102.2 (3)
C1—C2—S3	121.8 (3)	C27—C26—H26A	111.3
S4—C2—S3	114.8 (3)	C25—C26—H26A	111.3
C4—C3—C5	126.9 (4)	C27—C26—H26B	111.3
C4—C3—S1	116.3 (3)	C25—C26—H26B	111.3
C5—C3—S1	116.8 (3)	H26A—C26—H26B	109.2
C3—C4—C6	127.9 (4)	O4—C27—C26	126.0 (4)
C3—C4—S2	115.9 (4)	O4—C27—C22	126.9 (4)
C6—C4—S2	116.2 (3)	C26—C27—C22	107.1 (3)
C3—C5—H5A	109.5	C29—C28—C30	107.6 (3)
C3—C5—H5B	109.5	C29—C28—C25	111.8 (3)
H5A—C5—H5B	109.5	C30—C28—C25	114.1 (3)
C3—C5—H5C	109.5	C29—C28—C22	116.3 (3)
H5A—C5—H5C	109.5	C30—C28—C22	112.5 (3)
H5B—C5—H5C	109.5	C25—C28—C22	94.4 (3)
C4—C6—H6A	109.5	C28—C29—H29A	109.5
C4—C6—H6B	109.5	C28—C29—H29B	109.5
H6A—C6—H6B	109.5	H29A—C29—H29B	109.5
C4—C6—H6C	109.5	C28—C29—H29C	109.5
H6A—C6—H6C	109.5	H29A—C29—H29C	109.5
H6B—C6—H6C	109.5	H29B—C29—H29C	109.5
C8—C7—C9	124.6 (4)	C28—C30—H30A	109.5
C8—C7—S3	116.3 (4)	C28—C30—H30B	109.5
C9—C7—S3	119.1 (3)	H30A—C30—H30B	109.5
C7—C8—C10	125.6 (5)	C28—C30—H30C	109.5
C7—C8—S4	116.4 (3)	H30A—C30—H30C	109.5
C10—C8—S4	117.9 (4)	H30B—C30—H30C	109.5
C7—C9—H9A	109.5	C32—C31—S10	121.6 (3)
C7—C9—H9B	109.5	C32—C31—H31A	106.9
H9A—C9—H9B	109.5	S10—C31—H31A	106.9
C7—C9—H9C	109.5	C32—C31—H31B	106.9
H9A—C9—H9C	109.5	S10—C31—H31B	106.9
H9B—C9—H9C	109.5	H31A—C31—H31B	106.7
C8—C10—H10A	109.5	C31—C32—C33	110.3 (3)
C8—C10—H10B	109.5	C31—C32—C38	120.7 (3)
H10A—C10—H10B	109.5	C33—C32—C38	98.4 (3)
C8—C10—H10C	109.5	C31—C32—C37	119.4 (3)
H10A—C10—H10C	109.5	C33—C32—C37	101.9 (3)
H10B—C10—H10C	109.5	C38—C32—C37	102.7 (3)
C12—C11—S6	121.3 (3)	O8—C33—C34	126.1 (3)
C12—C11—S5	123.6 (3)	O8—C33—C32	126.3 (4)
S6—C11—S5	115.1 (3)	C34—C33—C32	107.5 (3)
C11—C12—S8	122.3 (3)	C33—C34—C35	101.5 (3)
C11—C12—S7	123.6 (3)	C33—C34—H34A	111.5
S8—C12—S7	114.1 (3)	C35—C34—H34A	111.5
C14—C13—C15	123.5 (4)	C33—C34—H34B	111.5
C14—C13—S5	117.3 (3)	C35—C34—H34B	111.5
C15—C13—S5	119.2 (3)	H34A—C34—H34B	109.3
C13—C14—C16	125.5 (4)	C34—C35—C36	105.8 (4)
C13—C14—S6	115.7 (3)	C34—C35—C38	103.2 (4)
C16—C14—S6	118.8 (3)	C36—C35—C38	103.7 (3)
C13—C15—H15A	109.5	C34—C35—H35	114.3
C13—C15—H15B	109.5	C36—C35—H35	114.3
H15A—C15—H15B	109.5	C38—C35—H35	114.3
C13—C15—H15C	109.5	C35—C36—C37	103.1 (3)
H15A—C15—H15C	109.5	C35—C36—H36A	111.1
H15B—C15—H15C	109.5	C37—C36—H36A	111.1
C14—C16—H16A	109.5	C35—C36—H36B	111.1
C14—C16—H16B	109.5	C37—C36—H36B	111.1
H16A—C16—H16B	109.5	H36A—C36—H36B	109.1
C14—C16—H16C	109.5	C36—C37—C32	104.2 (3)
H16A—C16—H16C	109.5	C36—C37—H37A	110.9
H16B—C16—H16C	109.5	C32—C37—H37A	110.9
C18—C17—C19	128.6 (4)	C36—C37—H37B	110.9
C18—C17—S7	115.9 (3)	C32—C37—H37B	110.9
C19—C17—S7	115.5 (3)	H37A—C37—H37B	108.9
C17—C18—C20	127.8 (4)	C39—C38—C40	107.9 (4)
C17—C18—S8	117.0 (3)	C39—C38—C35	114.2 (3)
C20—C18—S8	115.2 (3)	C40—C38—C35	112.0 (4)
C17—C19—H19A	109.5	C39—C38—C32	113.6 (3)
C17—C19—H19B	109.5	C40—C38—C32	114.9 (3)
H19A—C19—H19B	109.5	C35—C38—C32	94.0 (3)
C17—C19—H19C	109.5	C38—C39—H39A	109.5
H19A—C19—H19C	109.5	C38—C39—H39B	109.5
H19B—C19—H19C	109.5	H39A—C39—H39B	109.5
C18—C20—H20A	109.5	C38—C39—H39C	109.5
C18—C20—H20B	109.5	H39A—C39—H39C	109.5
H20A—C20—H20B	109.5	H39B—C39—H39C	109.5
C18—C20—H20C	109.5	C38—C40—H40A	109.5
H20A—C20—H20C	109.5	C38—C40—H40B	109.5
H20B—C20—H20C	109.5	H40A—C40—H40B	109.5
C22—C21—S9	122.1 (3)	C38—C40—H40C	109.5
C22—C21—H21A	106.8	H40A—C40—H40C	109.5
S9—C21—H21A	106.8	H40B—C40—H40C	109.5
C22—C21—H21B	106.8	H91—O9—H92	99 (3)
S9—C21—H21B	106.8	H101—O10—H102	107 (3)
H21A—C21—H21B	106.7	H111—O11—H112	107 (3)
C21—C22—C27	110.2 (3)	H121—O12—H122	102 (3)
C21—C22—C23	118.5 (3)	C1—S1—C3	96.2 (2)
C27—C22—C23	103.6 (3)	C1—S2—C4	96.6 (2)
C21—C22—C28	120.8 (3)	C2—S3—C7	96.3 (2)
C27—C22—C28	99.6 (3)	C2—S4—C8	96.2 (2)
C23—C22—C28	101.4 (3)	C13—S5—C11	96.0 (2)
C24—C23—C22	104.0 (3)	C11—S6—C14	96.0 (2)
C24—C23—H23A	111	C12—S7—C17	96.2 (2)
C22—C23—H23A	110.9	C12—S8—C18	96.8 (2)
C24—C23—H23B	110.9	O3—S9—O2	113.6 (3)
C22—C23—H23B	111	O3—S9—O1	112.2 (3)
H23A—C23—H23B	109	O2—S9—O1	110.6 (2)
C25—C24—C23	104.3 (3)	O3—S9—C21	104.2 (2)
C25—C24—H24A	110.9	O2—S9—C21	108.35 (19)
C23—C24—H24A	110.9	O1—S9—C21	107.39 (19)
C25—C24—H24B	110.9	O5—S10—O7	112.9 (2)
C23—C24—H24B	110.9	O5—S10—O6	113.7 (2)
H24A—C24—H24B	108.9	O7—S10—O6	110.3 (2)
C24—C25—C26	105.8 (4)	O5—S10—C31	103.8 (2)
C24—C25—C28	102.1 (3)	O7—S10—C31	107.88 (18)
C26—C25—C28	102.3 (4)	O6—S10—C31	107.68 (18)

### Hydrogen-bond geometry (Å, º)

**Table:**

*D*—H···*A*	*D*—H	H···*A*	*D*···*A*	*D*—H···*A*
C5—H5*A*···O5	0.96	2.37	3.263 (7)	155
C6—H6*C*···O2	0.96	2.57	3.472 (5)	156
C6—H6*A*···O5	0.96	2.49	3.450 (6)	173
C6—H6*B*···O1^i^	0.96	2.49	3.409 (5)	161
C19—H19*A*···O3^ii^	0.96	2.48	3.420 (6)	167
C19—H19*B*···O6^ii^	0.96	2.44	3.387 (5)	169
C20—H20*A*···O3^ii^	0.96	2.34	3.287 (7)	171
C26—H26*A*···O12^iii^	0.97	2.5	3.435 (7)	162
C34—H34*A*···O9^iv^	0.97	2.52	3.476 (6)	169
O9—H91···O10	0.89 (2)	1.86 (3)	2.729 (7)	166 (8)
O9—H92···O8	0.88 (2)	2.13 (3)	2.976 (6)	160 (6)
O10—H101···O6	0.85 (2)	1.95 (2)	2.795 (5)	177 (6)
O10—H102···O7^v^	0.84 (2)	2.04 (4)	2.815 (5)	154 (7)
O11—H111···O1^i^	0.86 (2)	2.01 (3)	2.821 (5)	158 (7)
O11—H112···O2	0.83 (2)	2.06 (3)	2.863 (5)	162 (7)
O12—H121···O11	0.87 (2)	1.92 (3)	2.732 (7)	156 (7)
O12—H122···O4^i^	0.88 (2)	2.15 (4)	2.965 (6)	154 (7)

Symmetry codes: (i) *x*+1, *y*, *z*; (ii) *x*, *y*+1, *z*; (iii) −*x*+1, *y*+1/2, −*z*+1; (iv) −*x*+1, *y*−1/2, −*z*+2; (v) *x*−1, *y*, *z*.
